# Human Development VI: Supracellular Morphogenesis. The Origin of Biological and Cellular Order

**DOI:** 10.1100/tsw.2006.255

**Published:** 2006-11-14

**Authors:** Søren Ventegodt, Tyge Dahl Hermansen, Trine Flensborg-Madsen, Maj Lyck Nielsen, Joav Merrick

**Affiliations:** ^1^ Quality of Life Research Center, Teglgårdstræ de 4-8, DK-1452 Copenhagen K, Denmark; ^2^ Research Clinic for Holistic Medicine, Copenhagen, Denmark; ^3^ Nordic School of Holistic Medicine, Copenhagen, Denmark; ^4^ Scandinavian Foundation for Holistic Medicine, Sandvika, Norway; ^5^ Interuniversity College, Graz, Austria; ^6^ Zusman Child Development Center Soroka University Medical Center Ben Gurion University of the Negev, Beer-Sheva, Israel; ^7^ National Institute of Child Health and Human Development, Jerusalem, Israel; ^8^ Office of the Medical Director Division for Mental Retardation Ministry of Social Affairs, Jerusalem, Israel

**Keywords:** holistic biology, theoretical biology, clinical holistic medicine, morphogenesis, ontogenesis, developmental biology, Denmark

## Abstract

Uninterrupted morphogenesis shows the informational potentials of biological organisms. Experimentally disturbed morphogenesis shows the compensational dynamics of the biological informational system, which is the rich informational redundancy. In this paper, we use these data to describe morphogenesis in terms of the development of supracellular levels of the organism, and we define complex epigenesis and supracellular differentiation. We review the phenomena of regeneration and induction of Hydra and amphibians, and the higher animals informational needs for developing their complex nervous systems. We argue, also building on the NO-GO theorem for ontogenesis as chemistry, that the traditional chemical explanations of high-level informational events in ontogenesis, such as transmutation, regeneration, and induction, are insufficient. We analyze the informational dynamics of three embryonic compensatory reactions to different types of disturbances: (1) transmutations of the imaginal discs of insects, (2) regeneration after removal of embryonic tissue, and (3) embryonic induction, where two tissues that normally are separated experimentally are made to influence each other. We describe morphogenesis as a complex bifurcation, and the resulting morphological levels of the organism as organized in a fractal manner and supported by positional information. We suggest that some kind of real nonchemical phenomenon must be taking form in living organisms as an information-carrying dynamic fractal field, causing morhogenesis and supporting the organisms morphology through time. We argue that only such a phenomenon that provides information-directed self-organization to the organism is able to explain the observed dynamic distribution of biological information through morphogenesis and the organism's ability to rejuvenate and heal.

## INTRODUCTION

The existing morphogenetic theory does not explain how cells recruit the information to organize the different structures of the embryo. Caused by a misunderstanding of the cell, the levels of the organism and cell determination and differentiation have not been explained. Our papers about ontogenesis try to change this fact by giving a new theory for the cell[1] and explain supracellular ontogenesis and the order in biological systems (in this paper). We have discussed the fact that it has not been possible to explain the powers of the cells by use of chemical gradient models or the existing theory of DNA as donor of all cellular information in order to make the cells able to organize the ontogenesis[2]. The development from a single undifferentiated cell, the zygote, to all the different types of cells that create complex tissues and organs, such as nerve, brain, muscle, eye, bone, or nail, has not yet been understood. The ability of the cells to communicate and orient themselves in the embryo and organism in order to enable formation and maintenance of the organs and the wholeness of the body has long been a complete mystery. In the mentioned papers[1,2], we give our explanation of these astonishing biological enigmas.

We chose to describe the mechanism behind the organization of the biological system at all levels as “information-directed self-organization”. This way, the living being can be described as an information-directed, self-organizing, complex, dynamic system (complex as combined/put together, dynamic as motion by itself), in this series of papers often referred to as, the complex dynamic.

We propose that cell determination and ontogenesis can be explained if cells at all developmental levels use *information-directed self-organization*. For example, the building of organelles, tissues, and organs is organized by cell recruitment of complex information that is supplied to the organelles, tissues, and organs through an information-carrying field. This can be illustrated, as an example, by a flower in development where the information-carrying fields develop different structures in an illustrative way (e.g.,[3] p. 38, Figs. 22–34).

We think the existence of information in biological systems is realized through the high degree of organization. From the beginning, the curiosity of the human being has aimed to understand this biological enigma, for instance, through an expression of a specific élan vital, a movement founded by the French philosopher Henri Bergson (1859–1941). As the fact that today these kinds of unwieldy ideas has been abandoned due to the idea that nature is ruled by “ordinary” and simple principles, and in spite of the discovery of DNA, it has not yet been possible for science to explain the mechanism behind the creation of living being’s morphogenesis.

Ontogenesis is often described by the use of nonparticular ideas as “morphological fields”[4] or “positional information”[4] that means, information given as a consequence of position in a developing biological system (e.g.[3] p. 38, Figs. 22–34). Of course, these relief concepts do not explain anything, but express that the formation of the patterns in biological systems apparently involves the whole spaciousness of the biological system, in a complex and incomprehensible way. In this paper, we discuss the supracellular terms of ontogenesis in the view of our hypothesis. In another paper[1], we described the ontogenesis of cellular development, but in this paper, we will discuss the supracellular conditions of ontogenesis.

## WHAT WE THINK THE CELL IS NOT

First, the cell is not just chemical machinery. The point of view of contemporary molecular biology is that the information that organizes the cell's inner order and structure, as well as the tissues, organs, and the form of the whole organism comes form the DNA of the organism's genome[4], makes this less likely. According to the C-value paradox, the beings of high evolutionary levels are more complex, but on the other hand, they do not have much more DNA than simple (including single-celled) organisms[4]. Humans only contain 300 times as much DNA as large bacteria, and only one-third compared to the Lily[4]. The macroevolution, the evolution of living being’s shape, seems to be independent of the microevolution, the evolution of molecular structures. For example, the DNA of human beings and chimpanzees are 99% identical. This is the same difference as seen between twin species of mammalians and fruit flies, respectively[4]. On the other hand, all kinds of vertebrates have the same fundamental building plane. For instance, they have identical (bones), muscles, organization of the brain, and so on[7], only the form is different. This means that it is highly questionable that the structure and organization should be directly related to the molecular functions, and with this, the existence of another explanation is more realistic. The problems concerning the understanding of information in biological systems have also been explained, without much luck, by the use of different mathematical interpretations[8,9]. But most scientists feel that there has not yet been a theory to explain the distribution of information in biological systems. The existing models simply do not explain the organization of the extreme complexity, stability, and reproduction related to interacting cells. This is also a concern for the chemical gradient models that fail to explain the development in biological organisms. These act on the idea that information in biological systems is directed through a chemical arrangement from the genes. Such models calculate how the information is transferred from genes to shape. These models are the subject of this paper.

## THE ONTOGENESIS OF THE SUPRACELLULAR LEVELS

In our earlier paper[1], Fig. 1 shows a generalized representation of morphogenesis as a complex bifurcation, where the different levels of organization are specified as M1, M2, etc. It is worthwhile to notice that the whole organism, in parallel with the gradual development of the complexity, is able to go through a radical reorganization of structure on all levels, as it is known from the metamorphosis of vertebrates (for instance, amphibians). In tadpoles, thyroidea hormones trigger the metamorphosis. If the thyroidea gland is removed, the result is a giant tadpole. This also goes through a process of metamorphosis to form ordinary frogs after injection of hormone[3]. In this way, the systems are seemingly uninfluenced by the size. When the giant tadpoles are much longer then 2 mm, it is not likely that morphological information is promoted through diffusion (for instance, as a reaction-diffusion structure).

### The Phenomenon of Compensation

#### *Transmutations and Positional Information through the Morphogenesis of Insects*

In a convincing way, transmutations[4] show that information can be found at tissue level, but not as expected at the cell level. The imaginable disk that normally develops to one of the fruit fly organs, for instance, a wing, is able to change its development spontaneously into a leg or an antenna. Also known from the fruit fly is the special, homeotic or atavistic mutations that give morphological disturbances resembling transmutations. In the opinion of some scientists, these mutations are interpreted as cell determination directed by the genes[4], but the mechanism behind this is not understood in molecular biological terms. What can be observed is that one complete structure replaces another. So it looks as if the whole package of positional information is exchanged[10]. However, we believe that this interpretation is doubtful. The imaginable disks can experimentally be shown to match with a morphogene field, an area of tissue that functions as a whole, concerning positional information (see below: the amphibian regeneration)[10].

#### *Phenomenon of Regeneration*

*Hydra* regeneration — positional information in a simple three-dimensional system: The little freshwater polyp, that is the most simple of all cnidarians, is assumed to be the first creature on earth with a nerve system.[4]. Its body wall contains three layers of tissue. The outer layer is the epidermis with sense cells.

The innermost layer is the gastrodermis with secretory and nutrient-absorbing cells. In between these two layers is a layer (mesogloea) traversed by an uncomplicated network of simple nerve cells[4,11]. This nerve tissue is able to send ring-shaped impulses in all directions along the nerve network from the contact point[12]. This makes *Hydra* capable of making complicated and un-understandable behavioral patterns. It is, for instance, able to catch water flies, swim, and make somersaults. If *Hydra* is separated in two, both parts regenerate immediately[13]. This means that *Hydra* can be compared to a morphogene field (see next section). A very good question is how a newly formed *Hydra* is able to control these patterns of movements. We think that this can only be explained by positional information.

The amphibian regulation and morphogene fields have been examined by scientists[14,15]. Parts of an amphibian embryo can be removed without any injury. If, for instance, an eye or another part of its brain is removed, the surrounding tissue compensates for this. This is called regulation. There is a limit to how big a part it is possible to take away without injuring the embryo. Early in the scientific history, a "morphogene field" was operationally defined as a part of the embryo inside which it is possible to make regulations.

Gradually as the embryo develops, it gets more and more fields that make up minor and major parts of the whole. This is the case for all vertebrates, but with mammals, the amounts of embryological fields reach the maximum. In the fetus area, inside the "field of limbs", tissue can be removed without disturbing the embryonic development, but in adults, it is only possible to remove tissue from the "field of the little finger tip". Gradually, as the amounts of cells expand through development, one area is divided into several[4]. This corresponds to an ongoing delivery from the biological system of information through positional information. In newts, limbs can grow out again through formation of a normal lower leg in a way that is directed by positional information. This is the case in spite of the fact that the over leg is turned surgically before the outgrowth[4]. Regeneration and metamorphosis show that morphological information in general is accessible, also after completion of morphogenesis.

## Phenomenon of Induction

To induce means to influence with each other. A long row of experiments have been carried out, e.g.[16,17] and interpreted as if the tissues in developing systems interacts on an abstract way that can be described as an induction or redefinition of positional information. A very good example of this is a total lack of development of the nervous system that results in a blockage of the invagination of the mesoderm through the morphogenesis[16]. Another classical example is the induction of the neuroectoderm of the eyes lens[17] that also gives a review over experiments that throw light on the information in biological systems. If the tissues are not positioned orderly compared to each other, the development stops, and the information can no longer be recruited from the biological system.

Early in this century it was shown that transplantation of a dorsal lip from one amphibian blastula to a ventral area of another one, could induce the formation of a smaller secondary embryo ventral in the last one. This was at first interpreted as if the information to the embryo was lying in this region. From this comes the expression, organizer region[15]. But this interpretation had to be rejected, because it turned out that many strange substances could have the same effects. Examples of this are other tissues – also boiled and fixed –, steroids, organic systems, and cotton with turpentine. Here the formation of sub-cellular patterns is induced[15] that can be described as a redefinition and duplication of the potential information of the system.

As described through all these examples, the distribution of information necessary for organizing the different levels of biological systems cannot be described in conventional terms. In our model, the biological system can be described as a Chinese box model of information transmitting interactions. Through cell morphogenesis the information of the biological system can manifest itself through positional information.

## The Morphogenesis of the Nervous System

### Morphogenesis of the Brain

The morphogenesis of the nervous system is, because of the enormous amount of information this needs, without any doubt the most impressive achievement of biological nature. Obviously, it is difficult to measure the amount of information that has to be used for this performance. However, anyone that studies a map of the housefly's brain[18] may be excited and astonished by its complexity. This brain only takes up 1/3 $mm^3$ and weigh 0.4 mg, but contains 340.000 of the most complex and varying neurons of the animal kingdom connected in ways that are immensely precise and variegating, as "in all probability one cell body may give rise to several sets of arborizations, which functions as separate integrative units"[18]. The details of the brain of a housefly, gives the impression of the existence of an extremely specific determined connectivity. And in accordance with this the housefly is able to do all neural functions from the beginning without learning. Today, the development of such a system can only be explained in conditions of transfer of information through positional information that is read by the cells and their axons and dendrites.

Contrary to insects and lower invertebrates, it is the case for lots of mammalians, especially humans, that the nervous system is developed further after birth – humans goes though a process called myelination at least until the child is two years old[19]. The human has a considerable ability to learn, but we know from experiments with apes that much perception and behavior are embedded in the biological information[16,20]. It is not known if this information is manifested through the connectivity or if it is just transferred to a functionally level of the nervous system. But apparently the cortical reproduction of the body (in maps) is not "hard wired", but able to go through momentary re-organization after nerve-racking. This means that the representation exists as functionally patterns in the brain[16], in agreement with the last of the two alternatives.

### Axon Growth In Vivo is Directed through Positional Information

Excrescence of axons in vitro: As earlier mentioned, the axon excrescence in vitro is influenced by interior factors. However, frequently the axon excrescence in vitro is dominated by the influence from outside factors – as for instance a sticky lane on the button of the Petri dish[4,15], or a gradient of NGF[4]. This has lead to an intensively *in vivo* research, after "epigenetic" factors – factors that are involved with the genetic regulation of developmental pathways, leading to the cell differentiation – through the evolution.

Concerning excrescence of axons in vivo, special invertebrate neurons can be recognized between different individuals, and develop almost the same axons and dendrites[21,22]. Also the exterior stimuli leading to the well-known grasshopper neurons, in which axons at first have an identical growth and since grow in different directions have been studied and concluded: "It is highly unlikely that the growth cones we have been studying are directed passively by mechanical guidance cues"[23].

In vertebrates, many examples are known concerning the precision of axon growth following complicated patterns not explainable by mechanical (or chemical) footprints. The retina of the gold fish is extremely precisely projected to tectum. This organization happens through addition of rings of retina through the development, while the tectum develops through regional outgrowth from the side. The impression of dynamics in this system is completely conformed by experiments. Here, the right retinotope mapping of the whole retina on the whole tectum is re-created. This was possible in spite of cutting of nerves, blockade of axon excrescence, and re-movement of tissue from retina and tectum etc.[4]. Through development of vertebrate limbs, the nerves can be observed to grow in complicated patterns that seem extremely determined. As for instance, the pattern of nerves in wings is almost completely symmetrical[4]. It is also possible to show that if a pair of spinal cord segments is turned around in an early stage of the development of a chicken, then the motor neuron of a specific segment still finds the right muscle group to enervate[4]. If a spinal cord segment is turned through the development of an amphibian, axons that usually grow caudal, suddenly first grow in a rostral manner, but soon after it turns and continue its growth in the normal cordally way[15].

We conclude that it is not yet understand what mechanism – seemingly extremely dynamic – that leads axons through the development. But generally it does not seem to be "epigenetic factors". The excrescence of axons, seemingly, can only be described by positional information. This means that they get the information to the organization, after their position in the developing organism.

## DISCUSSION

Potential information of the vertebrate development of limbs was reviewed[4,15] and accordingly in humans the buds of arms and legs are visible in the beginning of the 5$^{th}$ week, where these are established by a mesenchym-core covered by a layer of ectoderm. This forms, apically on the bud, the apical, ectodermal crest that seemingly orients the bud in a palmary – dorsally way. Most caudally on the bud exists an area that corresponds to the organizer region in amphibians. This is called the zone of polarizing activity or ZAP. If this is moved from a wing bud of a chicken, to a rosally area of another one, the result is supernumerary boons of underarms, hands, and fingers. But, as with the organizer region, here it has been shown that other tissues and matter, as for instance retinoic acid, can have the same effect. The results of these experiments can be described as a re-definition of the positional information in the morphological field of limbs. In other experiments a block of mesoderm, that is known to develop from a leg bud to the structure of a thigh, has been inserted apically on a wing bud. Such a manipulation gives a wing with a toe on the tip[4]. The information from the leg bud has therefore been preserved in an abstract way. Not unexpected, a leg structure was formed, that was adapted to the new position of the wing bud. A mechanical interpretation of this kind of experiments is very difficult, because the mesenchymic cells of the leg and wing apparently seem completely identical. And later, these cells, so far as is known, also have the same differential pathway and form the same type of cells. Thereby it looks as if the information does not lie at the cell level, but instead at the level of tissues. This phenomenon is called non-equivalence[4]. The information from the genes is manifested through that cells is able to read their position in the wholeness of the structures and are able to differentiate after this. In this way, the superior patterns that specifically characterize a finger or a wing can be formed.

Several experiments have been done concerning the development of limbs, and a lot of different results has appeared, but; "… These results must be telling us something about the biochemistry and cell biology of limb morphogenesis, but no one has yet discovered what"[15]. So, the mechanisms are unknown, but the description of the course of events happens in a way that can only be explained by positional information. Therefore scientists talk about evidence of positional information[15]. This also counts for the nerve system. I.e. since *Hydras* nerve net apparently does not have any superior structures, and a new-formed *Hydra* quickly begins to search for its food, we propose that its movements- and behavioral-patterns may come from the biological system through the same positional information as the one that gives its body its form. It seems reasonable to think that positional information plays a similar role in the development of all nerve systems, since simple mechanisms usually are the fundament for development of the more complicated ones, through the evolution.

### The Morphogenesis Operates through Complex Epigenesis

First the zygote is created. This proliferates to an amount of cells. These organize mutually, and are now able to organize the embryo, when the information as a pattern is carried on to the embryos whole from the inner levels through the information transformed interactions. The size of the embryo, puts (through the amount of cells and other things) a natural limit for the manifestation of information. Gradually as the embryo grows, the patterns are supplied so the patterns of information that fits the actual pattern of the embryo, is manifested through interaction. When the embryo takes a new structure, new patterns that fits with the embryo, can be adapted and so forth. In this way all patterns are supplied, gradually as it is possible, and the organism expands to more and more complex phases of its biological program.

This can be understood as a dissipative process that spreads the complexity out through the levels of the biological system. The direction in time of the development has been one of the biggest theoretical biological enigmas through time. Why does the morphogenesis never pace backwards? All physical equations (but the thermo dynamic, dissipative ones) can be reversed: A dissipative process in the information, a degeneration, a decay. Even if we observe the opposite, that something very simple as a fertilized egg develops to something very complex, the description of the morphogenesis through information-transmitting self-organization makes sense. This is the case because a dissipative process expands from the finer innermost to the coarse outermost levels through a dispersal of the complexity.

Information directed self-organization of elements, which themselves are formed through information directed self-organization can be called complex epigenesis. The transfer of new information demands the organization that earlier information transformation has resulted in. The real strength of the complex epigenesis is, that it allows some patterns of information to enter into the organelles, cells and supra cellular organizations. This is done in a way so these patterns of information – even if they are represented together at the finer levels – can be recruited independent of each other anyway. This happens through similarity between structures and patterns of information. So an extremely complex organism through the morphogenesis can fold out in a pattern that is extremely reproducible and firm.

Information transformation interactions in biological patterns may be connected to biological system's ability to utilize a characteristic of the matter, that is not normally noticed in the biological systems (compare – modern physical disciplines as the chaos theory[24]). Throughout the evolution this nature phenomenon has been cultivated and used. So maybe it is reasonable to suppose that this has made the life possible and derived the evolution by mastering the conditions of information in biological systems. Biological coherence giving rise to information transmitting biological interactions are a rather unexpected and seemingly fundamental phenomenon of living nature.

## The Information to the Organism may be Present in a Kind of Chinese Box System

The description of positional information is very different from an ordinary molecular system description, because it involves that the information exists as roomy extended patterns that are able to interact locally with the different parts. This means that the cells can orient themselves according to the positional information and the other way around. The positional information should be able to read the position of the cells and correct this if the embryo is disturbed in its growth, as we can see through regeneration. Consequently we propose the existence of information-transmitted interactions between the superior level, and the parts that makes this level: The whole body contra the tissue systems, these contra organs and these contra cells etc. The organization between cells is never tighter then needed. In the same way, the supra cellular levels are very badly organized. But the organs as well as the organisms whole, on the other hand are extremely nicely formed. This again shows the existence of information directed self-organization on the highest levels of organization.

The imaginable disk and the transmutations show that positional information in insects comes in packages that are only possible to be used in its whole. The regeneration in *Hydra*, seemingly, shows that the positional information that specifies the form is also responsible for the supply of information to the nerve system. In amphibians, new positional information through the development is organized in a way as a kind of Chinese box system of morphological fields created inside each other. In this way the superior levels keep specifying the lower ones. In our model, this corresponds to the existence of information-transmitting interactions between the different levels of the embryo. The induction trials shows that the tissues represents the positional information on a supra cellular level, and that information-transmitted interactions between tissues of the same level with individual, positional information seems to be a decisive trait of the morphogenesis.

Seemingly, morphogenesis cannot be understood neither as pre-formation nor as epigenesis[25], but if we regard it as complex epigenesis using *information-directed self-organization* it seems that we actually can understand it. The information directed self-organization implies that all information to the organism (eventually except the structure of the small molecules) that is absorbed by the organism, has to be present in the biological system – and evidently on fine, interior levels of the Chinese box system.

We therefore conclude that the biological system can be described as a Chinese box system of information transmitting interactions[1]. Through the ontogenesis and the morphogenesis of the cell and the supra cellular levels, the information of the biological system can manifest itself, as described in another paper[1] and this paper, through positional information.

## Criticism of our Ideas Concerning the Ontogenesis

Professor Claus Emmeche has given his comments to our discussion of the morphogenesis in the papers describing the ontogenesis[Claus Emmeche 1989 Personal contact] and below we will discuss these in the light of our hypothesis. He comments the idea that the same force coordinates all levels of organization in biological systems in an ordered fractal manner by describing a very busy day concerning Christmas shopping. He describes different levels of organization as: movements between people on the shopping street; movement of the arms holding out for different things; movement of the fingers holding out for the purse and the money in it. He thinks that this hardly involves that the coordinated ordered organization at a specific level should be dependent of the organization of the underlying level. We do not agree with this. Firstly, the body, arms, fingers movements, as a riverbank where it is possible to move deeper and deeper into finer and finer structures, describes a kind of fractal levels. Seemingly, the patterns of the single levels could be chaotic compared to each other. But if we compare with the butterfly effect, where lots of levels are involved from the butterfly itself to huge hurricanes, seen in the light of Henry Feigenbaum’s chaos theory, the result of the most disordered turbulence shows up to be very ordered, and the organization of the butterflies movements, are well coordinated with the turbulent movements of the hurricane – the hurricanes movements depends on the movements of the butterfly. So, it is hardly current to establish the absence of coordinated order and organization in biological systems (any systems) from this. If the organization of the different levels seems chaotic compared to each other, it could very well be a consequence of comparing the levels in the wrong perspective.

He also criticizes the assumption that an early transfer of information to the cytoskeleton may take place. He thinks that the cytoskeleton could be self-organized instead. In our analysis something global and “intelligent” has to deliver the information that mediates the dynamic organization of the cytoskeleton for the cell to move coordinated. The informational needed is more than the chemistry of the cytoplasm can create itself.

We believe that the ontogenesis demands a large amount of information; Claus Emmeche argues that this is a problematic view (Claus Emmeche 1989, personal contact). The problem is more how the information is delivered to the cells, more than how many bits of information can be compressed with a smart compressing algorithm.

## CONCLUSIONS

Our model describes the biological system as a Chinese box model of information transmitting interactions. The whole organism in parallel with the gradual development of the complexity is able to go through a radical reorganization of structure on all levels. At tissue level information can be found in a way where one complete structure replaces another, each corresponding to a morphogene field. We think e.g. *Hydra* makes up a morphogene field organized by positional information that generally is accessible before and after completion of the morphogenesis.

The morphogenetic development can be explained as transfer of information through positional information that is read by the cells and their axons and dendrites, but it is not yet understand what mechanism leads axons through this development. However, much perception and behavior are embedded in the biological information indicating that the representation exists as functionally patterns in the brain, but generally not as “epigenetic factors”. We conclude that the excrescence of axons only can be described by positional information. This means that the information to the organization is organized according to the position in the developing organism. We think that positional information plays a similar role in development of all nerve systems, since simple mechanisms usually are fundamental for development of more complex ones through the evolution.

The information from the genes is manifested in a way so the cells are able to read their position in the wholeness of the structures and differentiate in a way so superior patterns, as e.g. a finger or a wing, can be formed. We think such development can only be explained by positional information. Such information causes information directed self-organization of elements, which themselves are formed through information directed self-organization etc. Such development where transfer of new information demands the organization that earlier information transformation has resulted in what we call complex epigenesis. The strength of the complex epigenesis is that it allows some patterns of information to enter into the organelles, cells and supra cellular organizations. Biological coherence giving rise to information transmitting biological interactions are a rather unexpected and seemingly fundamental phenomenon of living nature.

We must therefore conclude that the biological system can be described as a Chinese box system of information-transmitting interactions, where the biological system is able to develop its form and maintain this throughout life. In spite of injuries this expanded system of information-transmitting interactions can always adjust all the parts of the organism in condition to each other and the fundamental building plane. All the levels interact with each other and the parts of each level interact, mutually. Thereby, morphological information from the finely divided levels in the cells can manage to be in force at the superior levels. On the other hand, the information from the superior levels, of cause, may reach through the levels and control the cell behavior, and thereby be able to regulate the gene expression. All these can be collected in the following description: Through the vertebrate morphogenesis a Chinese box system of morphological fields is established. Here the information-transmitting interactions between the levels and the single parts of the same level gives an enormous dynamic system, that is able to convey the information of the biological system, and maintain its form throughout the life.

The uninterrupted morphogenesis shows the potentials of the biological systems. The experimentally disturbed morphogenesis shows the compensational dynamics of the biological informational system (the informational redundancy). When we review the phenomena of regeneration and induction of *Hydra* and amphibians, and the higher animal’s informational needs for developing their complex nervous systems, we argue – also building on the NO-GO theorem for ontogenesis as chemistry[2] - that the traditional chemical explanations of high-level informational events in ontogenesis like transmutation, regeneration, and induction are most insufficient.

We have analyzed the informational dynamics of three embryonic compensatory reactions to different types of disturbances: 1) Transmutations of the imaginal disks of insects. 2) Regeneration after removal of embryonic tissue. 3) Embryonic induction, where two tissues that normally are separated experimentally are made to influence each other.

We found morphogenesis to be best described as a complex bifurcation, and the resulting many morphological levels of the organism as organized in a fractal manner and supported by positional information organized.

We suggest that some kind of real non-chemical phenomena must be taking the form in living organisms as an information-carrying dynamic fractal, causing morphogenesis and supporting the organism’s morphology through time. We argue that only such a phenomenon that provides information-directed self-organization to the organism is able to explain the observed dynamic distribution of biological information through morphogenesis and the organism’s ability to rejuvenation and healing.

## ACKNOWLEDGMENTS

These studies were supported by grants from IMK Almene Fond. Our research in quality of life has been approved by the Copenhagen Scientific Ethical Committee under number (KF)V.100.2123/91.
